# Treatment of Recurrent *Clostridium difficile* Infection in an Immunocompromised Patient with Severe Neutropenia Not Responding to Standard Therapy

**DOI:** 10.1155/2020/3089023

**Published:** 2020-02-25

**Authors:** Hajar AlQahtani, Saeed Baloch, Deanne Tabb

**Affiliations:** ^1^Department of Clinical Pharmacy Services, King AbdulAziz Medical City, Riyadh, Saudi Arabia; ^2^Department of Pharmacy Services, Piedmont Columbus Regional Healthcare System, Columbus, Georgia, USA; ^3^Department of Internal Medicine-Infectious Disease, Piedmont Columbus Regional Healthcare System, Columbus, Georgia, USA

## Abstract

One of the most effective strategies in reducing the risk of *Clostridium difficile* infection (CDI) recurrence is fecal microbiota transplantation (FMT). However, several adverse events have been reported post FMT, and data on the efficacy and safety of FMT in immunocompromised patients with hematological malignancies are rare. This report presents FMT treatment for refractory CDI in a severely immunocompromised patient. A 69-year-old female presented to the emergency department complaining of foul smelling, intractable, watery diarrhea and generalized abdominal pain. She was recently diagnosed with high-risk myelodysplastic Syndrome (MDS) requiring daily blood transfusions and reported multiple CDI episodes in the past treated successfully with metronidazole and vancomycin as mono- or combotherapy. During this admission, treatment with oral vancomycin (high dose) and intravenous metronidazole was unsuccessful, so FMT was administered. The patient recovered well despite an absolute neutrophil count (ANC) < 0.25 × 10^9^/L, and chemotherapy was initiated soon after. FMT was successful and safe in this patient, with no relapse and adverse events seen in 8 weeks of follow-up via phone calls and office visits.

## 1. Background

*Clostridium difficile* Infection (CDI) constitutes 20–30% of nosocomial antibiotic-associated diarrhea [1]. Only *C. difficile* producing toxins A and B are considered pathogenic and, therefore, clinically significant [2]. CDI is associated with a mortality rate between 7%–17% and an increased rate of 36%–58% for severe infections [3]. The clinical manifestations of *C. difficile*-associated diarrhea (CDAD) are mild-to-severe diarrhea to pseudomembranous colitis. Risk factors for CDAD are advanced age (>65 years of age), antibiotics exposure (clindamycin, 3rd generation cephalosporin, and fluoroquinolones), prolonged hospital stay (>4 weeks), immunocompromised status (cancer and chemotherapy), and use of histamine-2 blockers (H2B) and proton pump inhibitors (PPI) [4]. Limited antibacterial agents are available to treat CDI depending on infection severity including metronidazole, vancomycin, and fidaxomicin [4]. The real challenge with CDI is that 25% of patients will experience recurrence within 60 days after the initial episode. Moreover, the risk increases to 45% after the first recurrence and 75% after multiple recurrences [1, 4–6]. One of the most effective strategies in reducing the risk of CDI recurrence is fecal microbiota transplantation (FMT). FMT has been proven to be efficacious in many case reports, case series, retrospective studies, and even clinical trials for treatment of CDI [4]. The success of preventing recurrence with FMT is reported to be 70–100% [3]. Little data are available on the efficacy and safety of FMT in patients with hematological malignancies. We report a successful use of FMT in severe recurrent CDI not responding to intravenous (IV) metronidazole and high dose oral (PO) vancomycin in a severely immunocompromised patient with newly diagnosed Myelodysplastic Syndrome (MDS) with severe pancytopenia and refractory anemia requiring daily blood transfusion and immediate chemotherapy.

## 2. Case Presentation

A 69-year-old female presented to the emergency department complaining of intractable diarrhea, nausea, vomiting, generalized abdominal pain, and inability to eat or drink. She described her diarrhea as watery with a foul smell and excessive bowel movements (BMs) up to 10 times per day not responding to loperamide. She had been recently diagnosed with high-risk Myelodysplastic Syndrome (MDS) with severe pancytopenia and refractory anemia. Significant medical history included end-stage renal disease treated with hemodialysis three times weekly, congestive heart failure, diabetes, hypertension, hyperlipidemia, gastroesophageal reflux (on omeprazole 20 mg PO daily), and seizures. The patient also reported recurrent CDI, and this admission was her 5th episode (4th recurrence). According to the patient's electronic chart, previous episodes in 9/2017, 01/2018, and 09/2018 were treated with metronidazole and PO pulse taper vancomycin. FMT was planned in 2017 but not administered, and unfortunately, the patient was lost to follow-up from infectious disease after 2017. The patient had not yet received any chemotherapy for her MDS. The laboratory results shown in Table 1 revealed severe leukopenia, pancytopenia, neutropenia, anemia, and hypoalbuminemia. Her baseline liver function tests were normal except for albumin and septic work up was negative including blood and urine cultures. Stool sample was sent for C. diff testing which returned to be positive for C.diff antigen and toxins and negative for NAP1 strain.

## 3. Treatment

The patient received metronidazole 500 mg PO q 8 h for 48 hours without clinical improvement. Upon Infectious Disease (ID) consult, vancomycin 125 mg PO q 6 h was initiated and metronidazole was changed to IV. After 48 hours, the patient showed no improvement, which prompted the ID team to increase vancomycin dose to 500 mg. Again, with no clinical improvement noticed after vancomycin dose maximization, FMT was our last resort considering the patient's history, compromised immune status, and immediate need to start chemotherapy. Gastrointestinal, oncology, and other clinical services collaborated, and a date was established for the FMT procedure. In the meanwhile, the patient had begun to receive blood transfusions almost daily. A 24-hour vancomycin washout period was observed, and the patient continued IV metronidazole before the FMT procedure. During colonoscopy, 240 ml of microbiota preparation (OpenBiome item: FMP250, Somerville, Massachusetts, US) was injected into the cecum. Her ANC was 0.16 × 10^9^/L, which was the lowest recorded during her hospital stay. The endoscopy revealed inflammation and ulceration in the colon epithelial lining cells without pseudomembranous colitis seen. The patient tolerated colonoscopy very well and had no bowel movement the day after. We discontinued metronidazole based on the lack of evidence of pseudomembranous colitis during colonoscopy.

## 4. Outcome and Follow-Up

**Figure 1:**
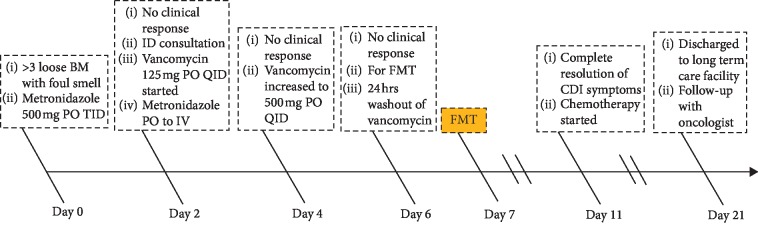
Patient hospital course timeline.

Watery diarrhea resumed the next day, but without foul smell. However, the patient continued to improve clinically and had no watery BM in subsequent days. After four days, she was able to receive and tolerate her first dose of chemotherapy treatment of decitabine. It is worth mentioning that she had been receiving vancomycin IV and meropenem for febrile neutropenia (FN), fluconazole, and valacyclovir for fungal and herpes simplex virus infections prophylaxis. Due to a seizure episode, meropenem was switched to aztreonam. Later on, IV antibiotics were changed to oral (PO) Bactrim for FN due to a severe allergy to fluoroquinolones and penicillin. Repeated blood cultures were negative. Finally, the patient was transferred to a rehabilitation facility to receive appropriate care. We followed her up for 8 weeks after FMT via phone calls and office visits and she has had no diarrhea since then (Figure 1).

## 5. Discussion

Patients with hematological malignancies are at higher risk for CDI secondary to multiple independent risk factors, including chemotherapy that disrupts intestinal normal flora and impairs intestinal epithelial cells conjunction, prolonged hospital stays, and antibiotics use [7, 8]. Multiple studies have identified certain chemotherapy agents that increase the risk of CDI such as platinum-, taxanes-, or anthracyclines-based chemotherapy regimens [8]. There are no data on the rate of CDI recurrence in hemo-oncologic patients; therefore, we expect that 25% of those patients will experience recurrence within 60 days after the initial episode, as seen in all CDI patients. The risk increased to 45% after the first recurrence and 75% after multiple recurrences in non-hemo-oncologic patient populations [1, 4–6].

Fuereder and his colleagues conducted the largest clinical study of CDI in hemo-oncology patients [8]. In this retrospective study, 144 hemo-oncologic patients with microbiologically confirmed CDI were compared to 144 age and sex-matched hemo-oncologic patients with no CDI. Both groups were evaluated for CDI risk factors including antibiotics exposure, type of disease, and chemotherapy received. Findings suggest that antibiotics exposure increases the risk of CDI by 2–3 fold, with 79% of CDI-positive patients vs. 67% of CDI-negative patients receiving an antimicrobial agent within four weeks prior to onset of diarrhea (OR = 2.26, *p*=0.038). Beta-lactam and cephalosporin exposure were found to be associated with a higher risk of CDI (53% vs. 42%; OR = 1.88, *p*=0.042 in CDI positive and CDI negative groups, respectively). Interestingly, fluoroquinolone exposure was comparable in both groups; therefore, the risk of CDI was similar (39.8% vs. 36.1%, respectively; OR = 1.33, *p*=0.319). Surprisingly, no association was identified between any specific chemotherapy agent and increased risk of CDI in this study.

A recent meta-analysis on FMT for the treatment of CDI in immunocompromised patients (including patients with solid and hematologic malignancies, human immune deficiency (HIV), organ transplants, and autoimmune diseases) showed that the resolution rate is 87% after the first FMTand 93% after the second FMT [5]. There was no significant analysis provided by the authors regarding information on the ANC at the time of FMT for patients with hematologic malignancies (*N* = 20), which is of great interest for this study.

Hefazi et al. reported on the efficacy and safety of FMT for recurrent CDI in 23 cancer patients treated with chemotherapy. In this retrospective study, 13 patients had hematologic malignancies. Only one of these patients had active disease and was on chemotherapy 4 weeks before FMT with an ANC = 0.49 × 10^9^/L on the day of FMT [7]. The rate of relapse was 8% (1/12) 22 months after FMT given during the administration of antibiotics and chemotherapy.

Oral vancomycin has become the standard of care for the treatment of primary/recurrent CDI [4]. Other treatment modalities include oral metronidazole for non-NAP1 strain and fidoxamicin. However, in some cases, none of these treatment approaches helps in treating or reducing the rate of CDI recurrence, especially in patients with higher risk for CDI due to active malignancies. This prompts clinicians to change their perspective and consider the role of the intestinal microbial community in preventing and reducing risk of CDI relapse. It is well known that oral therapy will not help in restoring the balance between beneficial and pathogenic intestinal normal flora.

FMT is an innovative method to restore intestinal normal flora and was used to treat the first documented CDI case by Schwan in 1983 [9]. It is also recommended by the Infectious Diseases Society of America (IDSA) guideline for treatment of subsequent recurrent CDI (3 episodes) [4]. FMT is associated with a high clinical success of 90%. Moreover, in immunocompromised patients, including patients with cancer, the resolution rate is 76–78% after the first FMT and 89–93% after a second/subsequent FMT [10, 11]. A more recent meta-analysis reported an even higher rate of clinical success (87%) after the first FMT and 93% after two or more FMTs [5]. The same study showed evidence of higher success rates after upper FMT (via endoscopy, capsules, and nasogastric tube) versus lower FMT (via endoscopy) (92% vs. 84%). The first randomized open label study that compares between single versus multiple FMTs was published in 2018. The goal was to compare between single FMT (FMT-S) followed by 14 days oral vancomycin versus multiple FMTs (FMT-M) every 3 days until disappearance of pseudomembranous followed by oral vancomycin. Authors conclude that FMT-M achieved significantly higher cure rates than FMT-S (100% vs. 75%, *p*=0.0) [12].

Despite these success rates, several adverse events have been reported post FMT, including aspiration pneumonia/pneumonitis following upper FMT or during sedation for colonoscopy, infections including bacteremia, abdominal pain, nausea, and irritable bowel syndrome (IBS). A recent guideline was published to provide guidance for practitioners to ensure quality control measures while handling FMT to prevent such adverse events [13].

The clinical question in our case was the safety of administering FMT in severely immunocompromised patients. Our patient had an ANC of <0.25 × 10^9^/L most days during hospitalization and 160 on the day of FMT. We were unsuccessful in finding an answer after conducting an extensive literature review. Therefore, we decided to use FMT in our patient given her advanced cancer and uncontrolled CDI.

In our case, FMT was used successfully to treat severe recurrent CDI in a patient with MDS with severe neutropenia. The patient tolerated FMT well and experienced remission immediately. The patient started chemotherapy which was tolerated, and no relapse was reported in 8 weeks of follow-up. We are planning to continue to follow her until 12 weeks given that the immunosuppression effect of chemotherapy can extend even after acute neutropenia for at least 12 weeks until B and T cells are replaced. In conclusion, this is the first case report that demonstrates the safety and efficacy of FMT for recurrent CDI in patient with severe neutropenia. Further prospective studies are necessary to prove safety of FMT for treatment of CDI in severely immunocompromised patients.

## 6. Learning Points/Take Home Messages

- *Clostridium difficile* Infection (CDI) recurrence occurs in approximately 25% of all cases, and severe cases are associated with high mortality.
- Fecal microbiota transplantation (FMT) is one of the most effective treatments for refractory CDI, but its safety, and efficacy in severely immunocompromised patients is not well documented.
- We report the successful use of FMT in a severely immunocompromised patient with myelodysplastic syndrome.

## Figures and Tables

**Table 1:** Laboratory results on admission.

Laboratory parameter	Result
WBCs (^∗^10^9^/L)	1.73
RBCs (^∗^10^12^/L)	2.96
Hgb (g/L)	8.6
Hct (%)	26.6
ANC (^∗^10^9^/L)	0.28
Lymphocytes (^∗^10^9^/L)	1.05
Monocytes (^∗^10^9^/L)	0.01
Scr (mg/dL)	16.90
Lactic acid (mmol/L)	1.8
Albumin (g/dL)	2.6
